# Supplementary figures and images for: Performance of Social Network Sensors during Hurricane Sandy
Source: PLoS One. 2015 Feb 18;10(2):e0117288. doi: 10.1371/journal.pone.0117288 (PMC4333288; doi:10.1371/journal.pone.0117288)

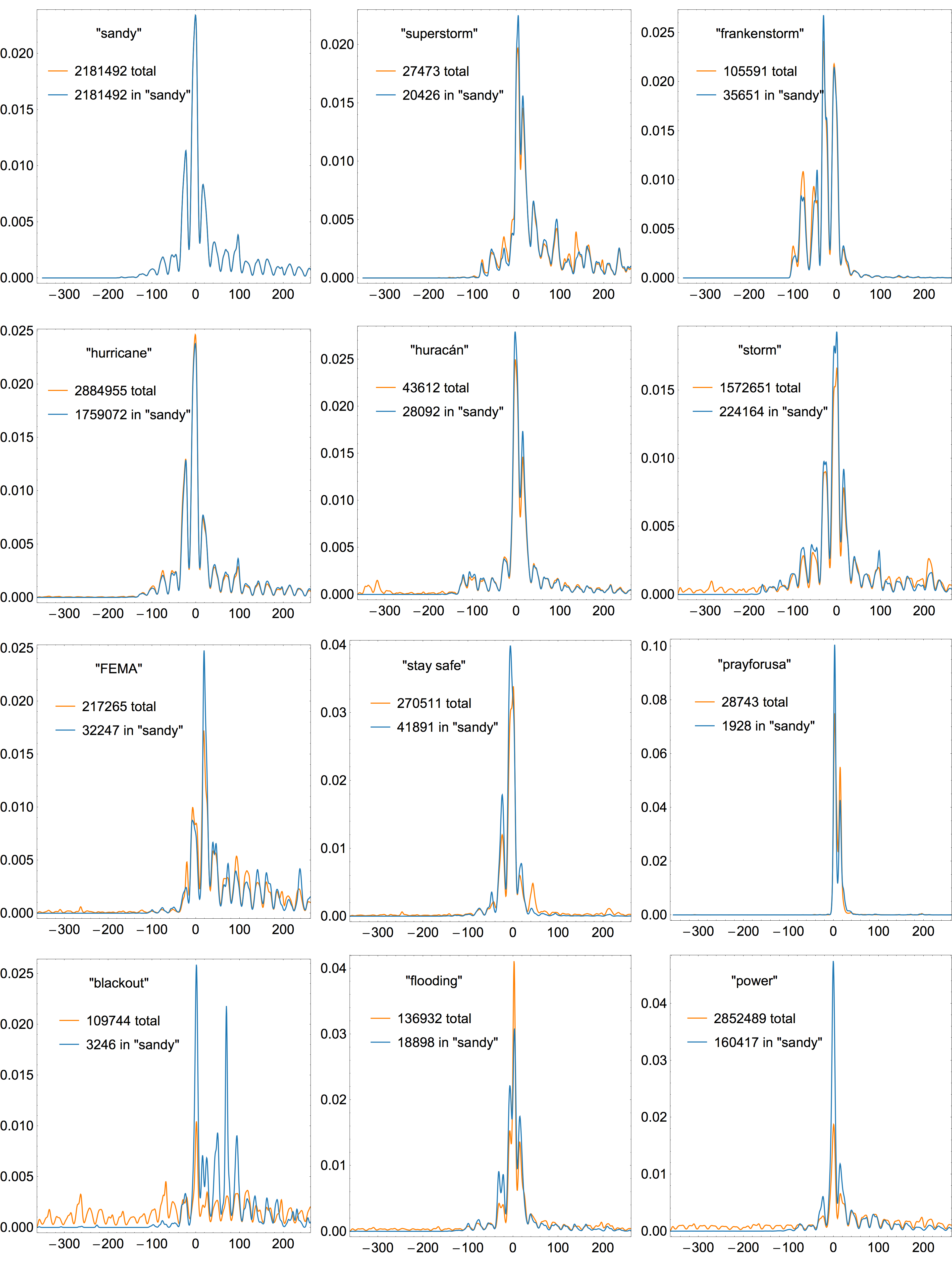

Supplement: S1 Fig — The figure shows the probability density functions for the occurrence of a keyword on its own in orange, and in combination with “sandy” in blue. The messages that occur before October 22 2012 (approximately at -200 hours on horizontal axis) are likely to be irrelevant to Hurricane Sandy and should be filtered out. Histograms are arranged in the approximate order of relevance. (TIFF) [file pone.0117288.s001.tiff]

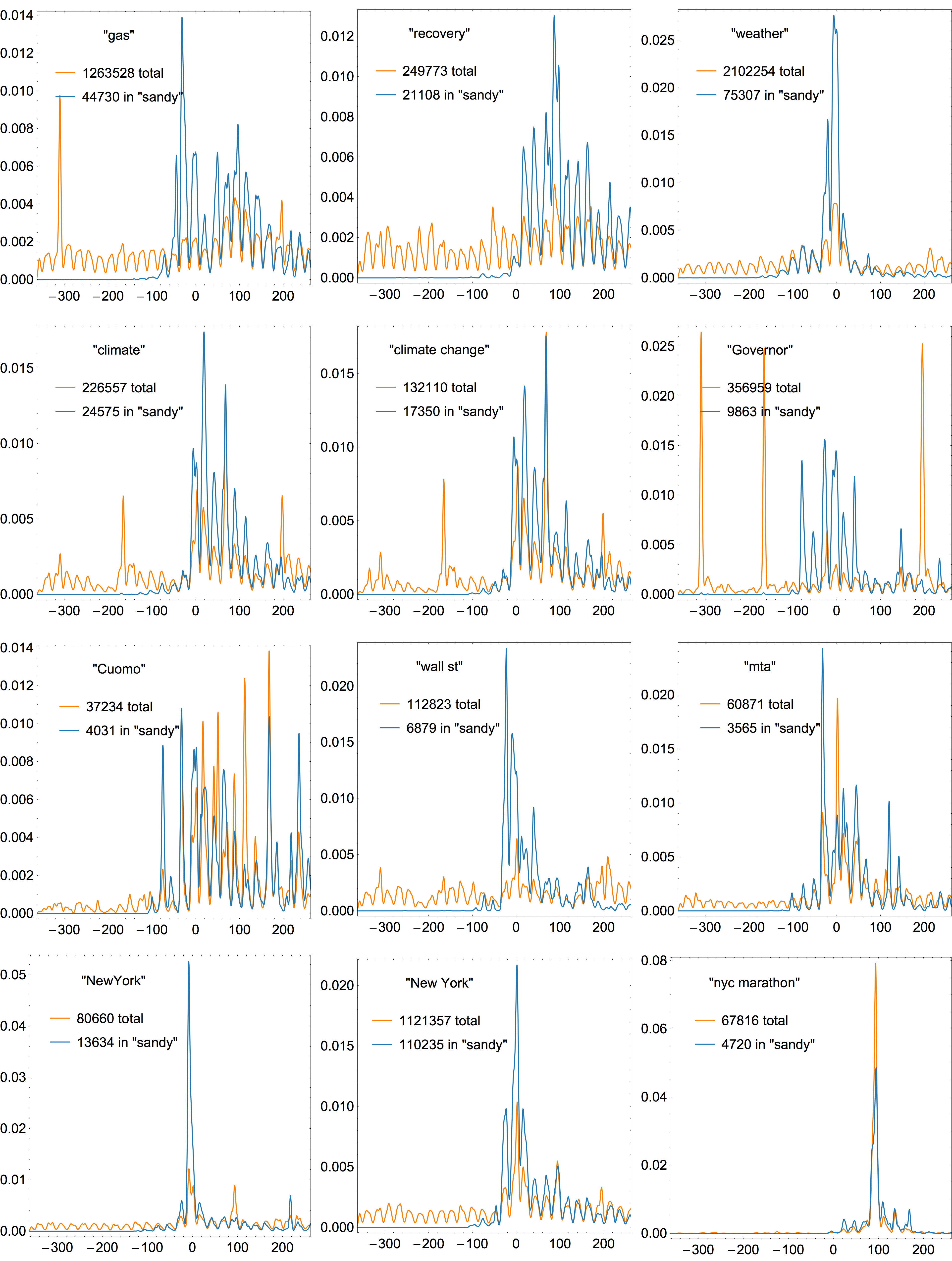

Supplement: S2 Fig — This figure continues the sequence from S1 Fig., with decreasing level of keyword relevance. Note the frequent incidence of use before October 22 2012. (TIFF) [file pone.0117288.s002.tiff]
