# Supplementary material for: Performance of Social Network Sensors during Hurricane Sandy
Source: PLoS One. 2015 Feb 18;10(2):e0117288. doi: 10.1371/journal.pone.0117288 (PMC4333288; doi:10.1371/journal.pone.0117288)
Supplement: S1 Table — There is an overlap in counts caused by the matches of a message to multiple keywords (e.g. a tweet with more than one keyword, like “widespread power losses after #sandy”; or a word “sandyaid” matching simultaneously to “sandyaid”, “sandy” and “sand”). However, the dataset itself contains only unique messages without duplication. (DOC) [file pone.0117288.s003.doc]

Table S1. List of keywords in the extended dataset accompanied by their corresponding message counts. There is an overlap in counts caused by the matches of a message to multiple keywords (e.g. a tweet with more than one keyword, like “widespread power losses after #sandy”; or a word “sandyaid” matching simultaneously to “sandyaid”, “sandy” and “sand”). However, the dataset itself contains only unique messages without duplication.

| Keyword | Count |  | Keyword | Count |  | Keyword | Count |
| --- | --- | --- | --- | --- | --- | --- | --- |
| sand | 4 846 422 |  | blackout | 213 520 |  | gás | 18 818 |
| power | 4 825 717 |  | franken | 210 277 |  | wallst | 18 423 |
| sandy | 4 745 099 |  | mta | 206 504 |  | nopower | 13 660 |
| hurricane | 4 680 290 |  | frankenstorm | 205 467 |  | stock exchange | 11 840 |
| weather | 3 333 025 |  | NewYork | 195 078 |  | Con Edison | 11 321 |
| storm | 2 555 196 |  | nyc marathon | 102 838 |  | comfortablysmug | 9 963 |
| New York | 2 348 535 |  | Cuomo | 92 014 |  | 911buff | 9 521 |
| gas | 1 991 524 |  | prayforusa | 91 293 |  | wallstreet | 8 855 |
| hurricane sandy | 1 906 749 |  | superstorm | 70 274 |  | new-york | 8 691 |
| hurricanesandy | 518 492 |  | nyse | 69 213 |  | opsafe | 7 714 |
| Governor | 498 135 |  | ConEd | 47 749 |  | Governor Cuomo | 6 786 |
| stay safe | 484 732 |  | huracan | 40 357 |  | sandy aid | 3 048 |
| recovery | 431 591 |  | wtc | 38 567 |  | pray for usa | 1 891 |
| climate | 420 217 |  | climatechange | 38 365 |  | operation safe | 1 482 |
| huracán | 371 157 |  | staysafe | 38 094 |  | trading floor | 1 130 |
| FEMA | 329 789 |  | ConEdison | 30 080 |  | sandy pets | 929 |
| flooding | 264 132 |  | conedison | 30 080 |  | StockExchange | 181 |
| no power | 261 998 |  | sandypets | 28 300 |  | op safe | 61 |
| climate change | 236 009 |  | nycmarathon | 26 179 |  | GovernorCuomo | 59 |
| wall st | 233 411 |  | sandyaid | 19 897 |  | TradingFloor | 21 |
