# Supplementary material for: Performance of Social Network Sensors during Hurricane Sandy
Source: PLoS One. 2015 Feb 18;10(2):e0117288. doi: 10.1371/journal.pone.0117288 (PMC4333288; doi:10.1371/journal.pone.0117288)
Supplement: S2 Table — (DOC) [file pone.0117288.s004.doc]

Table S2. Average activities and (messages per user), entry times $\left\langle t_{C}\right\rangle$ and $\left\langle t_{S}\right\rangle$, and lead-times $\Delta t$ (in hours) for control and sensor groups formed without location restrictions.

| Sample size |  |  | $\Delta t\pm\sigma(\Delta t)$, h | $\left\langle t_{C}\right\rangle$, h | $\left\langle t_{S}\right\rangle$, h |
| --- | --- | --- | --- | --- | --- |
| 500 | 3.23 | 8.65 | -10.9 ± 4.26 | 11.2 | 0.25 |
| 1000 | 3.17 | 8.07 | -9.74 ± 3.14 | 10.7 | 0.99 |
| 2500 | 3.24 | 7.34 | -8.93 ± 2.14 | 10.7 | 1.81 |
| 5000 | 3.20 | 6.63 | -8.06 ± 1.36 | 10.7 | 2.61 |
| 10000 | 3.20 | 5.97 | -7.30 ± 0.90 | 10.8 | 3.51 |
| 25000 | 3.22 | 5.13 | -6.18 ± 0.61 | 10.8 | 4.63 |
| 50000 | 3.20 | 4.61 | -5.37 ± 0.34 | 10.8 | 5.42 |
| 100000 | 3.21 | 4.16 | -4.68 ± 0.27 | 10.9 | 6.21 |
