# Supplementary material for: Performance of Social Network Sensors during Hurricane Sandy
Source: PLoS One. 2015 Feb 18;10(2):e0117288. doi: 10.1371/journal.pone.0117288 (PMC4333288; doi:10.1371/journal.pone.0117288)
Supplement: S3 Table — (DOC) [file pone.0117288.s005.doc]

Table S3. Average activities and (messages per user), entry times $\left\langle t_{C}\right\rangle$ and $\left\langle t_{S}\right\rangle$, and lead-times $\Delta t$ (in hours), with both control and sensor groups formed from users affected by the hurricane: “Control In – Sensor In” sampling.

| Sample size |  |  | $\Delta t\pm\sigma(\Delta t)$, h | $\left\langle t_{C}\right\rangle$, h | $\left\langle t_{S}\right\rangle$, h |
| --- | --- | --- | --- | --- | --- |
| 500 | 4.10 | 11.4 | -13.8 ± 4.29 | -0.07 | -13.9 |
| 1000 | 4.13 | 10.3 | -12.8 ± 2.64 | -0.18 | -12.9 |
| 2500 | 4.10 | 8.87 | -11.3 ± 1.86 | 0.08 | -11.2 |
| 5000 | 4.13 | 8.01 | -9.80 ± 1.31 | -0.05 | -9.85 |
| 10000 | 4.12 | 7.03 | -8.53 ± 0.92 | -0.05 | -8.59 |
| 25000 | 4.12 | 6.05 | -7.08 ± 0.60 | 0.02 | -7.06 |
| 50000 | 4.11 | 5.45 | -5.90 ± 0.36 | -0.03 | -5.92 |
| 100000 | 4.10 | 5.00 | -4.85 ± 0.25 | 0.03 | -4.83 |
