# Supplementary material for: Performance of Social Network Sensors during Hurricane Sandy
Source: PLoS One. 2015 Feb 18;10(2):e0117288. doi: 10.1371/journal.pone.0117288 (PMC4333288; doi:10.1371/journal.pone.0117288)
Supplement: S4 Table — (DOC) [file pone.0117288.s006.doc]

Table S4. Average activities and (messages per user), entry times $\left\langle t_{C}\right\rangle$ and $\left\langle t_{S}\right\rangle$, and lead-times $\Delta t$ (in hours) for control groups affected and sensors unaffected by the hurricane: “Control In – Sensor Out” sampling.

| Sample size |  |  | $\Delta t\pm\sigma(\Delta t)$, h | $\left\langle t_{C}\right\rangle$, h | $\left\langle t_{S}\right\rangle$, h |
| --- | --- | --- | --- | --- | --- |
| 500 | 4.07 | 7.73 | 5.14 ± 4.29 | 0.08 | 5.21 |
| 1000 | 4.15 | 7.43 | 5.15 ± 3.23 | 0.12 | 5.27 |
| 2500 | 4.10 | 6.78 | 6.07 ± 1.88 | 0.05 | 6.12 |
| 5000 | 4.10 | 6.28 | 6.37 ± 1.38 | -0.03 | 6.33 |
| 10000 | 4.11 | 5.74 | 6.88 ± 1.06 | -0.08 | 6.79 |
| 25000 | 4.11 | 4.98 | 8.03 ± 0.65 | -0.00 | 8.03 |
| 50000 | 4.11 | 4.48 | 8.98 ± 0.50 | -0.01 | 8.97 |
| 100000 | 4.11 | 4.03 | 10.1 ± 0.31 | -0.04 | 10.0 |
