# Supplementary material for: Performance of Social Network Sensors during Hurricane Sandy
Source: PLoS One. 2015 Feb 18;10(2):e0117288. doi: 10.1371/journal.pone.0117288 (PMC4333288; doi:10.1371/journal.pone.0117288)
Supplement: S5 Table — (DOC) [file pone.0117288.s007.doc]

Table S5. Average activities and (messages per user), entry times $\left\langle t_{C}\right\rangle$ and $\left\langle t_{S}\right\rangle$, and lead-times $\Delta t$ (in hours) for control groups unaffected and sensors affected by the hurricane: “Control Out – Sensor In” sampling.

| Sample size |  |  | $\Delta t\pm\sigma(\Delta t)$, h | $\left\langle t_{C}\right\rangle$, h | $\left\langle t_{S}\right\rangle$, h |
| --- | --- | --- | --- | --- | --- |
| 500 | 2.86 | 11.9 | -26.2 ± 4.08 | 15.8 | -10.4 |
| 1000 | 2.87 | 10.9 | -25.7 ± 3.16 | 16.0 | -9.69 |
| 2500 | 2.91 | 9.88 | -24.9 ± 2.06 | 15.8 | -9.06 |
| 5000 | 2.85 | 9.03 | -24.2 ± 1.34 | 16.0 | -8.22 |
| 10000 | 2.87 | 8.19 | -23.2 ± 1.01 | 15.8 | -7.44 |
| 25000 | 2.86 | 7.05 | -22.1 ± 0.53 | 15.8 | -6.31 |
| 50000 | 2.87 | 6.34 | -21.3 ± 0.36 | 15.8 | -5.52 |
| 100000 | 2.87 | 5.66 | -20.4 ± 0.28 | 15.8 | -4.67 |
