# Supplementary material for: Performance of Social Network Sensors during Hurricane Sandy
Source: PLoS One. 2015 Feb 18;10(2):e0117288. doi: 10.1371/journal.pone.0117288 (PMC4333288; doi:10.1371/journal.pone.0117288)
Supplement: S6 Table — (DOC) [file pone.0117288.s008.doc]

Table S6. Average activities and (messages per user), entry times $\left\langle t_{C}\right\rangle$ and $\left\langle t_{S}\right\rangle$, and lead-times $\Delta t$ (in hours) for both groups unaffected: “Control Out – Sensor Out” sampling.

| Sample size |  |  | $\Delta t\pm\sigma(\Delta t)$, h | $\left\langle t_{C}\right\rangle$, h | $\left\langle t_{S}\right\rangle$, h |
| --- | --- | --- | --- | --- | --- |
| 500 | 2.88 | 6.88 | -8.14 ± 4.36 | 15.7 | 7.52 |
| 1000 | 2.86 | 6.64 | -7.95 ± 3.05 | 15.9 | 7.91 |
| 2500 | 2.87 | 6.09 | -7.23 ± 2.05 | 15.8 | 8.59 |
| 5000 | 2.87 | 5.59 | -6.23 ± 1.41 | 15.6 | 9.40 |
| 10000 | 2.89 | 5.03 | -5.46 ± 0.98 | 15.7 | 10.3 |
| 25000 | 2.86 | 4.38 | -4.57 ± 0.61 | 15.8 | 11.3 |
| 50000 | 2.87 | 3.97 | -3.97 ± 0.38 | 15.8 | 11.8 |
| 100000 | 2.86 | 3.61 | -3.36 ± 0.25 | 15.8 | 12.5 |
